# Who Is He?

**Published:** 1887-05

**Authors:** 


WHO IS HE?—The *American Medical Journal* very truly remarks: There are thousands of men in this world who have a name only to live, but are dead —dead to the world and to all mankind, except it be to only a few who, through the busy din of life, happen to stumble upon them. They identify themselves with nothing; they plod the silent paths of darkness and obscurity, as if they had no object in life, no good to accomplish, no purpose in their being. They drop their bodies in the dust, and but few know of their death because they were not identified with the living and active, and their loss creates not even a ripple upon the placid surface of waters.

"But," says the reader of this note, "what has this to do with the medical profession?" It has everything to do. Do you not know that there are thousands of physicians in this country who have lost their identity? Go, ravel over the country, as I have done, and you will find a doctor here and there whom you have never heard of before. He has never written a word for any medical journal, never been identified with any County, State or National Association; never even become acquainted with his neighbor physicians, except that some kick or cuff has brought him to his senses. His whereabouts is unknown. Why? Because he has lost his identity. He has become indifferent to himself and to his own interests, and cares but little for the interests of others.

I would not want to live by any profession and isolate myself in this way. Let the profession know where you live. Be an Allopath, a Homœopath, an Eclectic, be something. Identify yourself with the society in which you move, at home and at large. Join your county society; if none, make one. Join your State Medical Society and pay your dues promptly, for it will pay you in the end. Identify yourself with the National Association, and you will be rewarded. Take at least one medical journal. Write an article, though short and broken it be—at least sign your name and address to a postal card, and let it be known that you live, move and have a being. By so doing you obtain an enviable position and a legitimate reputation that will do you good and that will live long after you are dead.

